# Analysis of Illicit Street Heroin Seized in Pakistan by Gas Chromatography–Mass Spectrometry (GC‐MS) for Identification of Adulterants and Impurities

**DOI:** 10.1155/ianc/4301930

**Published:** 2026-03-01

**Authors:** Muhammad Usman, Yawar Baig, Abid Naseer, Donatella Nardiello, Maurizio Quinto

**Affiliations:** ^1^ Department of Agriculture, Food, Natural Resource, and Engineering (DAFNE), via Napoli 25, I-71122, Foggia, Italy; ^2^ Narcotic Unit, Punjab Forensic Science Agency, Old Multan Road Thokar Niaz Baig, Lahore, 53700, Pakistan; ^3^ Department of Chemical Sciences, University of Catania, Viale Andrea Doria 6, Catania, 95122, Italy, unict.it; ^4^ Narcotics Forensic Laboratory, Anti-Narcotics Force Academy, H-11, Islamabad Capital Territory, Islamabad, Pakistan

**Keywords:** adulterants in street heroin, heroin, street drugs, street drugs in Pakistan

## Abstract

Heroin abuse poses a significant public health and law enforcement challenge globally, with concerns in Pakistan due to the prevalence of illicit street heroin and limited data on its composition. This study analyzed 706 heroin samples seized between 2017 and 2022 using gas chromatography–mass spectrometry (GC‐MS) and Fourier transform infrared spectroscopy (FTIR) to determine their chemical composition. The results revealed that street heroin in Pakistan is extensively adulterated with a variety of pharmaceutical agents, including acetaminophen, caffeine, diazepam, and dextromethorphan. Pareto analysis demonstrated that a small subset of adulterants accounted for most of the street samples, with caffeine, acetaminophen, and diazepam present in nearly 90% of analyzed samples. Impurities from incomplete synthesis, particularly 6‐monoacetylmorphine (97%) and acetylcodeine (95%), were also widespread. Some samples contained only adulterants, lacking heroin entirely. The widespread adulteration and presence of pharmacologically active impurities significantly increase the risk of toxicity and complicate clinical management of overdoses. These findings highlight the urgent need for drug monitoring and regulation, as well as targeted public health interventions. The comprehensive chemical characterization of street heroin provides valuable insights for forensic investigations, policy development, and epidemiological surveillance to address the evolving risks associated with illicit heroin use in Pakistan.

## 1. Introduction

Heroin is a semisynthetic derivative of morphine, a constituent of natural poppy resin. In Pakistan, it is the most widely abused drug after hashish. Due to its analgesic effect, which is two to three times more than its parent molecule (morphine), it causes dependence and addiction quickly. Heroin is classified as a Schedule I substance as per the United Nations Office on Drugs and Crime (UNODC) Single Convention on Narcotic Drugs, 1961 [1], due to its high potential for abuse and severe addictive properties [2–4]. Illicit street heroin is typically sold in an impure state, often containing a range of added substances that alter its potency and bulk [5]. In addition to the toxicity of illicit heroin, these adulterants can also be harmful. Due to the varying concentrations of the pharmacologically active substances in the street heroin, its use is frequently associated with acute toxicity and deaths due to overdose [6–8].

Impurities and adulterants in heroin can be broadly classified into three categories, i.e., naturally co‐extracted alkaloids, synthetic byproducts, and intentionally added substances. Naturally occurring opium alkaloids, such as codeine [9], morphine [9], papaverine [9], and noscapine [9], may remain in the final product when purification steps are inadequate, indicating crude or small‐scale processing methods. Synthetic impurities, such as acetylcodeine [10] or 6‐monoacetylmorphine [11], are formed during incomplete acetylation of morphine and can serve as chemical markers for the manufacturing route. Adulterants, however, are deliberately added to modify heroin’s appearance, potency, or bulk. These include pharmacologically active agents, such as caffeine [12], acetaminophen [13], phenobarbital [14], and benzodiazepines [7], as well as inert diluents, such as lactose [15] or sucrose [16]. The specific combination and proportion of these substances provide valuable insight into local trafficking patterns and adulteration trends [17]. Consequently, systematic identification of both impurities and adulterants serves as a key forensic tool for assessing heroin purity [18], tracing its origin [19], and evaluating associated health risks [20].

Drug profiling of heroin includes the analysis of impurities, diluents, and adulterants using analytical techniques, providing useful information for tactical (linking two or more samples) and strategic (determining geographic origin) intelligence investigation [21]. Identification of alkaloid impurities and substances found in illicit street drugs helps in the comparison of samples. The comparative studies of street heroin performed in several countries worldwide not only guide about the origin or geographic locality in which the drug is synthesized but also give information about the route of transportation/trafficking [11, 22–25].

For the analysis of heroin, different analytical techniques are being used for both research and forensic purposes. Quantitative and qualitative analysis of illicit drugs is carried out through various techniques which include capillary electrophoresis (CE) [26], nuclear magnetic resonance spectroscopy (NMR) [27, 28], gas chromatography–mass spectrometry (GC‐MS) [29–32], gas chromatography–flame ionization detector (GC‐FID) [33, 34], liquid chromatography–tandem mass spectrometry (LC‐MS/MS) [35, 36], Fourier transform infrared spectroscopy (FTIR) [5], Raman spectroscopy [37], and near‐infrared spectroscopy (NIR) [38].

In Pakistan, there is currently limited information available on the presence of adulterants and the purity of street heroin, which highlights the need for this study. This study aimed to report the impurities and adulterants in street heroin in Pakistan. The samples were seized by the law enforcement agency (Anti‐Narcotics Force [ANF]) and submitted to the Narcotics Forensic Laboratory, ANF Academy for analysis. GC‐MS was used as the primary analytical technique for the analysis of samples, due to its high sensitivity, specificity, and reliability for identifying heroin and its adulterants. FTIR was additionally employed to provide rapid, nondestructive confirmation of functional groups and chemical composition in the samples analyzed. This study presents a comprehensive overview of the chemical composition of street heroin in Pakistan and contributes to the broader understanding of the adulteration trends in the region.

## 2. Analytical Procedure

### 2.1. Standards and Solvents

All chemicals and standards were of purity above 95% and purchased from Fisher Scientific (USA) unless specified. Heroin standard (1 mg/mL in acetonitrile) was purchased from Cerilliant (USA). Acetylcodeine, codeine, diazepam, dextromethorphan, 6‐monoacetylmorphine, morphine, phenobarbital, and thebaine were purchased from Cerilliant (USA). Methanol (ACS grade) was purchased from Fisher Scientific (USA). Caffeine, acetaminophen, papaverine, concentrated sulfuric acid, and formaldehyde (38%) were purchased from Sigma‐Aldrich (USA). A 0.5‐µm PTFE syringe filter was purchased from Agilent Technologies (USA).

### 2.2. Seizure Data and Samples for Analysis

Data related to the seizures of heroin, its precursors, and adulterants were collected from the official website of the ANF, Pakistan (https://www.anf.gov.pk) for the period 2017–2022 dated on March 30, 2023 [39]. Among these seizures conducted by ANF, a total of 706 street heroin samples were submitted to the Narcotics Forensic Laboratory, ANF Academy, Pakistan, for narcotic content confirmation. These same samples were analyzed in this study to determine the adulterants and impurities in street heroin.

### 2.3. Sample Preparation

For each sample, 2 mg of a homogeneous mixture of illicit heroin powder was dissolved in 2 mL of methanol. After sonication and filtration through a 0.5‐µm PTFE syringe filter, 1 µL was injected into the GC‐MS system of analysis as reported earlier [40]. For fabric and other types of similar contents, approximately 5–100 mg of the sample was soaked in approximately 5 mL methanol, and the extract was filtered through a 0.5‐µm PTFE syringe filter, where the filtrate was concentrated to up to 1 mL which was further analyzed as mentioned previously [40].

### 2.4. Sample Analysis

All samples were preliminarily screened through colorimetric reactions using the Mecke reagent test and the Marquiss reagent test [41]. For analysis, Agilent 7890 B GC coupled with 5977 MSD and 7693A autosampler (Agilent Technologies, Palo Alto, CA) was used. The sample was injected at 20 psi pressure and 250°C temperature using split mode with a split ratio of 20:1. Helium was used as carrier gas at 20 psi and 0.5 mL/min. The Agilent DB‐5 column was held at 150°C for 1 min and increased at the rate of 25°C/min to 300°C for 5 min. The solvent delay was 2.50 min. The coupled MS has single quadrupole detector with MSD in EI mode (electron‐ionization source), source temperature of 230°C, and quad temperature of 150°C. Scan time segment starts with 43 amu and ends with 550 amu. The results of GC‐MS analysis of street samples were confirmed by reference standards and by comparing with the reported literature [41]. Illicit samples and standards were analyzed at the same parameters on GC‐MS. Sample blanks were run before each sample and quality control (QC‐positive and QC‐negative). The method was validated as per the UNODC and Scientific Working Group for the Analysis of Seized Drugs (SWGDRUG) [42, 43] method validation guidelines for specificity/selectivity, limits of detection (LOD), precision under repeatability and reproducibility, and matrix effect [40]. A GC‐MS method for qualitative identification of controlled substances was validated utilizing 39 diverse certified reference materials, samples, plant material extracts, and pharmaceutical tablets (randomly selected from casework), given in Supporting Table S1. Specificity and selectivity were evaluated through comparative spectral matching against established libraries (SWGDRUG2016, NIST05a), confirming unambiguous analyte detection across all samples and certified reference materials. LODs were established for acetaminophen, caffeine, codeine, diazepam, morphine, thebaine, cannabinol, cannabidiol, tetrahydrocannabinol, and heroin via serial dilutions from 500 to 5 μg/mL, defining reliable identification as signal‐to‐noise ratio ≥ 3, library match quality ≥ 85%, and the presence of major ions. Method precision was assessed through ten replicate analyses at concentrations spanning 1.25 − 2 × LOD, while matrix effects were investigated using complex mixtures, i.e., mix‐1 (heroin, diazepam, thebaine, and pheniramine), mix‐2 (heroin, caffeine, acetaminophen, and diazepam), mix‐3 (morphine, thebaine, papaverine, and alprazolam), mix‐4 (diazepam, thebaine, papaverine, and pheniramine), and mix‐5 (tetrahydrocannabinol, thebaine, papaverine, and diazepam) at approximate LOD levels.

GC‐MS validation outcomes demonstrated excellent specificity and selectivity, with all 39 samples yielding clear positive identifications. LOD values were determined as: 15 μg/mL for cannabidiol, cannabinol, and thebaine; 25 μg/mL for heroin; 10 μg/mL for diazepam, tetrahydrocannabinol, codeine, and caffeine; 100 µg/mL for acetaminophen; and 125 μg/mL for morphine, confirming appropriate sensitivity for trace‐level detection. Reproducibility was robust, evidenced by 100% successful identifications in replicate analyses with no false negatives. Matrix interference testing across all adulterated mixtures produced no false negatives, underscoring method reliability in complex mixtures and street samples. Optimal library combinations resolved spectral matches for all analytes, establishing the GC‐MS protocol appropriate for the identification of narcotics and psychotropic substances.

Four samples were randomly selected from a total of 706 samples to verify GC‐MS results and were analyzed on FTIR. This subset was chosen to represent different visual appearances to assess whether surface characteristics affected spectral features. Selected illicit samples and standards were analyzed on Thermo Nicolet iS10 FTIR coupled with diamond ATR. Approximately 10 mg of sample was placed on ATR assembly with the aperture set at 80 for wavenumber range from 4000 cm^−1^ to 650 cm^−1^ with resolution 4.000, for 32 scans [44]. Analytical standards were selected based on their documented prevalence as adulterants or impurities in heroin samples, as reported in profiling studies and recommended by the UNODC [45].

### 2.5. Statistical Analysis

The qualitative data from GC‐MS were subjected to descriptive statistics, frequency, and correlation analyses to identify the most common adulterants. The Pareto analysis (80/20 rule) was applied to identify the substances that contributed most frequently to the heroin samples, highlighting the key adulterants responsible for the majority of adulterants in street samples [46–48]. Pearson’s correlation was used to assess relationships between major constituents. Microsoft Excel 365 and JMP Pro 16 were used for statistical analysis, and data visualization was performed using Microsoft Excel 365, RStudio 2024.04.0, and ArcGIS 10.

## 3. Results and Discussion

### 3.1. Heroin and Precursor Seizure Data

The data related to the seizure of heroin, its precursors (i.e., morphine, poppy resin, and acetic anhydride), and some adulterants depict an alarming situation across the country. According to the available data [39], an average of 5414 kg/year of heroin was seized by the law enforcement authorities (i.e., ANF) over a five‐year period from 2017 to 2022. In addition to the heroin, large quantities of precursors used in its synthesis were also confiscated. Morphine, with an average seizure of 3356 kg/year, is a key precursor used in heroin synthesis. Opium (natural source of morphine), with an average seizure of 11361 kg/year, is another important precursor that is obtained from poppy plants. Acetic anhydride, a chemical used for the acetylation of morphine, was seized in large quantities, with an average of 1976 L/year confiscated over the same five‐year period. Furthermore, some benzodiazepines, i.e., diazepam, clonazepam, and tramadol, which are being used as cutting agent in the street heroin, were also seized [39]. The large quantities of seized heroin, its precursors, and some adulterants reflect the persistent trafficking and possible synthesis in clandestine laboratories. This highlights the availability of essential precursors and the difficulty of controlling clandestine heroin production [49]. The details of year‐wise seizure data are given in Table 1.

**TABLE 1:** Seizure data of heroin, its precursors, and some diluents.

Year	Heroin	Morphine	Opium	Acetic anhydride	Diazepam
2017	19754.6	7132.2	32574.1	917.5	735
2018	934.857	831	5321.65	4145	3000
2019	881.155	1015	1941.58	19.06	0
2020	4457.856	5976	6249.475	2037	0.247
2021	3415.987	3292.5	11044.67	570	1360
2022	3043.252	1893	11035.613	4173	6560
Total	**32487.707**	**20139.7**	**68167.088**	**11861.56**	**11655.247**

*Note:* Bold values indicate the grand total of seizures for each substance from 2017 to 2022. Additionally, ANF seized clonazepam tab. 85230 (2021‐2022) and tramadol 5200 (2022). Amount of heroin, morphine, opium in kilograms, acetic anhydride in liters, diazepam, clonazepam, and tramadol number of tablets. Source (Anti‐Narcotic Force official website: (https://www.anf.gov.pk) [39].

### 3.2. Physical Appearance and Colorimetric Reactions

**FIGURE 1:**
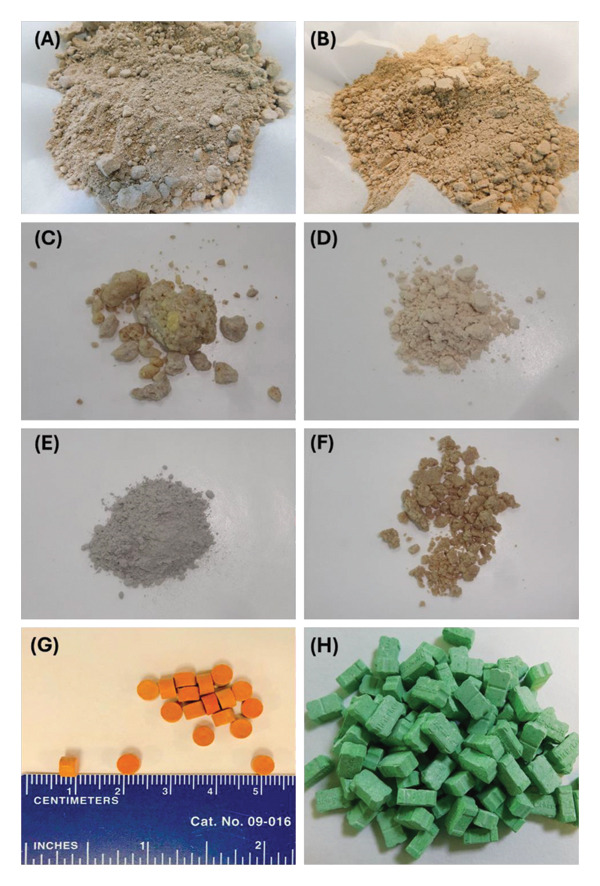
Physical appearance of some of the heroin samples analyzed: (A) Street heroin powder, (B) street heroin powder, (C) street heroin powder, (D) street heroin powder, (E) street heroin powder, (F) street heroin powder, (G) illicit morphia tablets containing CAF, DEX, acetyl codeine, hydromorphone, PAP, and NOS along with heroin., (H) illicit tablets containing heroin, methamphetamine, and adulterants found in street heroin, i.e., CAF, DEX, DIA, and 6‐MAM.

The physical state of the heroin samples was in the form of powder, which exhibited colors ranging from off‐white to light brown as reported in other countries, such as Denmark, Italy, and the United States [7, 18, 30]. The texture of the powder was homogeneous, without any visible impurities or particles on most occasions as shown in Figure 1. The variation in color could be due to the difference in various types of adulterants, their relative concentrations, and the origin or purity of heroin, i.e., the presence of poppy alkaloids. These characteristics could be important for identifying and distinguishing the samples. In the local drug market, a less frequently encountered form of heroin is available in the form of orange‐colored tablets referred to as “morphia tablets” [40]. Along with these tablets, cloth samples were also submitted, which had been soaked in the solution of heroin and dried. The cloth samples are likely intended for international trafficking purposes. In a few cases, a small amount of fine white, insoluble powder was visible; this was most probably inorganic in origin. This study had not investigated the inorganic composition of heroin samples as similarly not reported by other studies like Barnfield et al. [50].

The colorimetric reactions showed positive results for samples containing heroin and opium alkaloid impurities (*n* = 692), and all samples (*n* = 706) were further analyzed on GC‐MS for confirmation. Qualitative results for all 706 samples are provided in Supporting Table S2, which includes the raw peak areas for all analyzed samples with detected adulterants and impurities.

### 3.3. Qualitative Analysis Using GC‐MS

**FIGURE 3:**
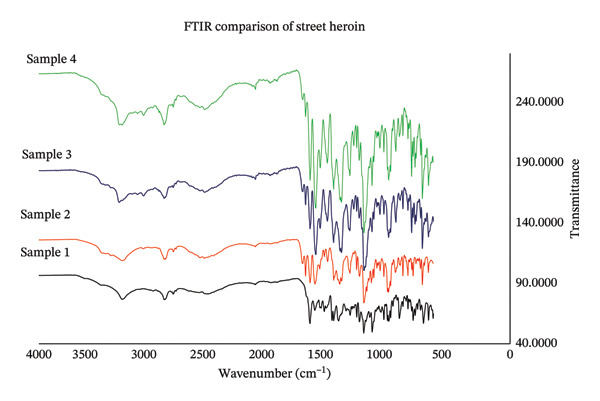
FTIR spectra of randomly selected street heroin samples.

The results of GC‐MS analysis showed that the street heroin is available with variable composition, having different pharmaceutical drugs in addition to heroin and poppy alkaloids. GC‐MS analysis confirmed twenty‐two substances, i.e., acetaminophen (ACE), caffeine (CAF), diazepam (DIA), dextromethorphan (DEX), chloroquine (CHQ), clotrimazole (CLO), nicotinamide (NIC), etiracetam (ETR), ketamine (KET), tramadol (TRA), chlorpheniramine (CHP), phenobarbital (PHE), lidocaine (LID), levorphanol (LEV), codeine (COD), morphine (MOR), papaverine (PAP), noscapine (NOS), hydrocodone (HCD), hydrocotarnine (HCT), acetylcodeine (ACC), and 6‐monoacetylmorphine (6‐MAM) including heroin in street samples. Among these twenty‐two substances, five substances, i.e., 6‐MAM, ACC, CAF, ACE, and DIA, were the common substances identified individually including adulterants and impurities in most of the samples analyzed. All the compounds, number, and percentage of their individual appearance in 706 samples are given in Table 2. The GC–MS total ion chromatogram (TIC) illustrating the major adulterants and alkaloids detected in street heroin samples is presented in Figure 2. FTIR analysis on randomly selected four samples (Figure 3) showed principal peaks of heroin at wave numbers, 1760 ± 5 and 1730 ± 6 cm^−1^ due to C=O stretching, while 1240 ± 5, 1170 ± 5, 1215 ± 5, and 909 ± 5 cm^−1^ are due to C‐O and O‐H functional group absorption.

**TABLE 2:** Adulterants and impurities as identified by GC‐MS analysis of street heroin.

Component	Description	Appeared in number of samples (percentage)
*ADULTERANTS IDENTIFIED*		
Acetaminophen	Analgesic drug	545 (77.19)
Caffeine	Stimulant	673 (95.33)
Chloroquine	Antimalarial drug	1 (0.14)
Chlorpheniramine	Antihistamine drug	1 (0.14)
Clotrimazole	Antifungal drug	20 (2.80)
Dextromethorphan	Cough suppressant drug	145 (20.54)
Diazepam	Anticonvulsant drug	385 (54.53)
Etiracetam	Antiepileptic	1 (0.14)
Ketamine	Analgesic drug	1 (0.14)
Levorphanol	Analgesic drug	1 (0.14)
Lidocaine	Local anesthetic drug	8 (1.13)
Nicotinamide	Vitamin B group	4 (0.57)
Phenobarbital	Anticonvulsant	1 (0.14)
Tramadol	Analgesic drug	6 (0.85)

*IMPURITIES IDENTIFIED*		
Acetylcodeine	Impurity due to acetylation of codeine	674 (95.47)
6‐Monoacetylmorphine	Impurity due to acetylation of morphine	685 (97.02)
Codeine	Opium alkaloid	36 (5.10)
Hydrocodone	Semisynthetic opioid	1 (0.14)
Hydrocotarnine	Opium alkaloid	17 (2.41)
Morphine	Opium alkaloid	30 (4.25)
Noscapine	Opium alkaloid	290 (41.08)
Papaverine	Opium alkaloid	297 (42.09)

FIGURE 2GC‐MS—total ion chromatogram showing adulterants in street heroin samples; 1: ACE, 2: CAF, 3: DIA, 4: ACC, 5: heroin, 6: PAP, 7: MOR, 8: 6‐MAM.

**Figure:**
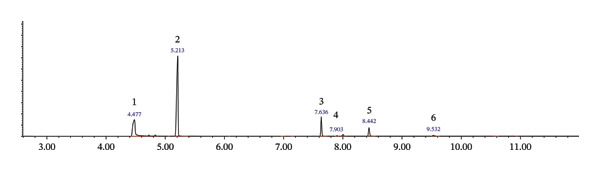
(a)

**Figure:**
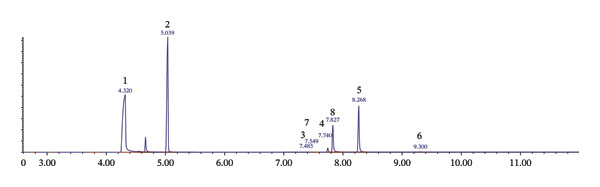
(b)

### 3.4. Most Common Adulterants in Heroin Samples

**FIGURE 4:**
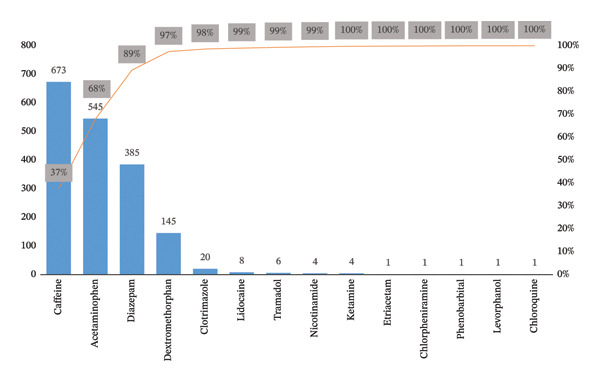
Cumulative percentage occurrence of adulterant in street heroin samples, based on Pareto analysis (80/20 rule) (Supporting Table S3).

Among the twenty‐two substances given above, fourteen compounds, i.e., ACE, CAF, DIA, DEX, CHQ, CLO, NIC, ETR, KET, TRA, CHP, PHE, LID, and LEV, are pharmacologically active drugs and are adulterated to increase the volume and mask the low concentration of heroin. The Pareto analysis (80/20 rule), in terms of cumulative percentage, highlighted the key adulterants in the street heroin that make up most occurrences (Supporting table S3). In Figure 4, cumulative percentage curve (orange line) represents the cumulative impact of each substance in terms of total occurrences across the 706 samples. CAF, ACE, DIA, and DEX were the top four most frequent substances in the samples analyzed, and they contributed heavily to the cumulative percentage. CAF was present in 673 samples, representing cumulative percentage of around 37% of the total substances analyzed. ACE follows with 545 occurrences, raising the cumulative percentage to about 68%. DIA, present in 385 samples, brought the cumulative total to 89%. By including DEX (145 occurrences), the cumulative percentage was approximately 97%. These four substances alone accounted for 97% of the adulterants in the heroin samples and illustrated a clear “Pareto effect” where a small number of adulterants contributed to the majority of the street heroin samples. The substances, such as CLO, LID, TRA, NIC, and KET, have contributed slightly, and the cumulative percentage gradually increased to 100%. Each of these lower frequency substances (such as PHE and CHQ) appeared in less than 10 samples and has little overall impact, but their presence can still be important for understanding variability in heroin adulteration.

Adulterants, such as ACE, CAF, DEX, and DIA, are added to heroin for various reasons, primarily to enhance the effect as it mostly contains low concentration of heroin. Each adulterant has some advantages for both drug dealers and users. For example, CAF is a stimulant that also enhances the vaporization of heroin at low temperature and improves the uptake of heroin [51]. CAF has also been one of the most frequently reported adulterants worldwide and has been reported in countries including Austria [12], Belgium [52], Egypt [53], Iran [9], Malaysia [31], the United Kingdom [54], the United States [55], and some other countries [5, 7, 10, 13–16, 18, 35, 51, 56–67], indicating its use as adulterant in global street heroin. ACE is added due to its analgesic effects and close melting point to that of heroin. The bitter taste of ACE masks low‐quality street heroin powder [68] and also reported in other countries, such as Australia [16, 64], Germany [66], Iran [9], Italy [5, 18, 51], Switzerland [15, 56], and others [7, 12–14, 31, 35, 52, 53, 57–60, 62, 65, 67] as given in Table 3. DEX is an opioid with dissociative properties and included to create altered states of consciousness or to imitate the effects of heroin and has been reported in Iran [9], Italy [5, 18, 51], Malaysia [31], Switzerland [15, 56], and Russia [59, 60], where dextromethorphan has been identified as a common additive in heroin, likely used for its central nervous system effects and to enhance perceived potency. DIA, a benzodiazepine, has also been observed in Denmark [7], Iran [9], Ireland [13], and Kuwait [14], indicating a regional and international pattern of benzodiazepine adulteration aimed to enhance the depressing effect of heroin due to its sedative properties [18, 31, 68].

**TABLE 3:** Adulterants and impurities reported in street heroin in some countries.

Sr	Adulterants and impurities	Country	Ref.
1	Acetaminophen, caffeine, chloroquine, and phenolphthalein	Afghanistan	[63]
2	Acetaminophen, caffeine, codeine, morphine, 6‐monoacetylmorphine, acetylcodeine, sucrose, glucose, and mannitol	Australia	[16, 64]
3	Acetaminophen, caffeine, cocaine, lidocaine, lactose, noscapine, and papaverine	Austria	[12]
4	Acetaminophen, caffeine, codeine, morphine, noscapine, papaverine, acetylcodeine, monoacetylmorphine, diacetamate, and methacetin,	Belgium	[52]
5	Codeine, 3‐monoacetylmorphine, 6‐monoacetylmorphine, and acetylcodeine	China	[69]
6	Acetaminophen, caffeine, griseofulvin, diazepam, phenobarbital, piracetam, methaqualone, procaine, barbital, ascorbic acid, salicylic acid, mannitol, glucose, and lactose/maltose	Denmark	[7]
7	Acetaminophen, caffeine, ephedrine, chlorpheniramine, phenobarbitone, methylenedioxymethamphetamine, theophylline, monoacetylmorphine, acetylcodeine, morphine, papaverine, and meconin	Egypt	[53]
8	Acetaminophen, caffeine, mannitol, lactose, saccharose, and meconin	France	[65]
9	Acetaminophen, caffeine, phenobarbital, methaqualone, nicotinamide, procaine, phenolphthalein, and salicylic acid	Germany	[66]
10	Acetaminophen, caffeine, methaqualone, oxazepam, ketazolam, nordazepam, pinazepam, and alprazolam	India	[67]
11	Acetaminophen, caffeine, noscapine, dextromethorphan, phenobarbital, diazepam, chloroquine, codeine, morphine, papaverine, acetylcodeine, and monoacetylmorphine,	Iran	[9]
12	Acetaminophen, caffeine, triacetin, diazepam, tramadol, mannitol, and sorbitol	Ireland	[13]
13	Acetaminophen, caffeine, methorphan, lidocaine, phenacetin, dimenhydrinate, diphenhydramine, metronidazole, aminopyrine, procaine, chloroquine, cocaine, diacetamate, 3‐hydroxy‐2,3‐dihydromaltol, hydroxy methyl furfural, menthol, phenoxyethanol, squalene, mannitol, acetylcodeine, codeine, morphine, thebaine, noscapine, meconin, papaverine 3‐monoacetylmorphine, 6‐ monoacetylmorphine, and triacetyl normorphine,	Italy	[5, 18, 51]
14	Acetaminophen, caffeine, diazepam, phenobarbital, alprazolam, papaverine, acetylcodeine, and monoacetylmorphine	Kuwait	[14]
15	Acetaminophen, caffeine, codeine, morphine, papaverine, noscapine, meconin, acetylcodeine, and monoacetylmorphine	Luxembourg	[57, 58]
16	Acetaminophen, caffeine, dextromethorphan, codeine, morphine, acetylcodeine, and monoacetylmorphine	Malaysia	[31]
17	Codeine, morphine, noscapine, acetylcodeine, and monoacetylmorphine	North Korea	[11]
18	Acetaminophen, caffeine, dextromethorphan, diazepam, hydromorphone, papaverine, noscapine, and acetylcodeine	Pakistan	[40, 70]
19	Caffeine, dextromethorphan, tolycaine, acetaminophen, methyl salicylate, pelargon, salicylic acid, meconin, codeine, papaverine, noscapine, morphine, hydrocotarnine, acetylcodeine, and monoacetylmorphine	Russia	[59, 60]
20	Caffeine, chloroquine, piracetam, acetylcodeine, and monoacetylmorphine	Singapore	[10, 61]
21	Morphine, meconin, papaverine, noscapine, acetylcodeine, and monoacetylmorphine,	Slovenia	[71]
22	Acetaminophen, caffeine, piracetam, and ocfentanil	Spain	[35, 62]
23	Acetaminophen, caffeine, dextromethorphan, gluconic acid, inositol, griseofulvin, glucose, mannitol, lactose, sucrose, fructose, papaverine, noscapine, phenacetin, acetylcodeine, and monoacetylmorphine,	Switzerland	[15, 56]
24	Caffeine, barbiturate, procaine, strychnine, morphine, acetylcodeine, and monoacetylmorphine	The United Kingdom	[54]
25	Acetaminophen, caffeine, aminopyrine, diltiazem, diphenhydramine, dipyrone, levamisole, phenacetin, quetiapine, quinine, procaine, lidocaine, morphine, noscapine, papaverine, acetylcodeine, and monoacetylmorphine	The United States	[55]

### 3.5. Co‐occurrence of Different Adulterants in Street Heroin

CAF was the most common adulterant added in the street heroin and was found in 95 percent of illicit street samples in combination with others. However, in 6.6 percent of the total samples, CAF was found to be the only principal adulterant. ACE is the second and DIA the third most common adulterants, with 77.19 and 54.53 percent, respectively, of the total samples analyzed. DIA was observed in one street sample as a single adulterant. While ACE was not observed as a single adulterant in any sample, it always appeared in combination with other adulterants. The other CLO, LID, TRA, NIC, KET, ETR, CHP, PHE, LEV, and CHQ are less common adulterants (Table 2).

**FIGURE 5:**
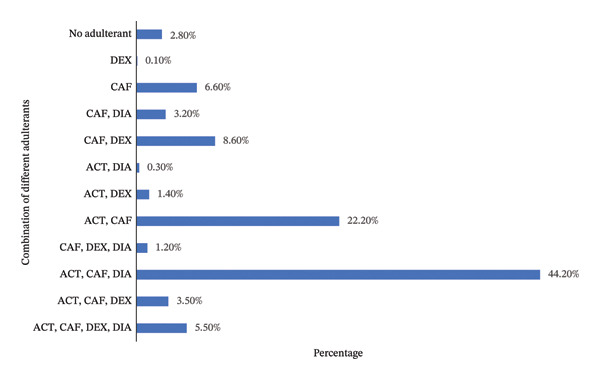
Frequency of different combinations of adulterants in the analyzed street samples. ACT, CAF, and DEX were present in 44.20% of the total samples analyzed and found to be the most common combination of adulterants.

The most common combination of adulterants was CAF, ACE, and DIA, and this combination was observed in 312 (44.19%) of 706 samples analyzed. Simultaneously, CAF and ACE were found in 157 (22.23%) samples, while CAF and DEX were found in 61 (8.64%) samples, with no other cutting agent besides the ones mentioned. All combinations of different adulterants in street heroin observed during the analysis of 706 samples are given in Figure 5.

### 3.6. Impurities From Precursor Opium and Synthesis

As heroin is synthesized from the poppy resin from *Papaver somniferum*., several impurities were observed in street heroin. There are several impurities that come from the poppy resin, either impure MOR was used or through incomplete acetylation of MOR or due to side reactions, and these include COD, MOR, PAP, NOS, ACC, and 6‐MAM. Some of these mentioned impurities, i.e., COD, MOR, PAP, and NOS, are the constituents of the natural poppy resin naturally occurring alkaloids found in opium, which is used to produce heroin [72]. These impurities have been reported globally, reflecting comparable production processes and raw material sources. For instance, COD has been identified in heroin samples from Australia [16, 64], Belgium [52], China [69], Iran [9], Italy [5, 18, 51], Luxembourg [57, 58], Malaysia [31], North Korea [11], and Russia [59, 60], while MOR has been commonly detected in countries, such as Egypt [53], Iran [9], Italy [5, 18, 51], and the United States [55]. PAP and NOS have been reported in countries including Austria [12], Belgium [52], Iran [9], Italy [5, 18, 51], Russia [59, 60], and Switzerland [15, 56], supporting their origin from incompletely refined poppy extracts. ACC is produced due to the acetylation of COD during heroin synthesis, while 6‐MAM is produced due to incomplete acetylation of MOR. Both ACC and 6‐MAM have been frequently observed in international seizures, reported in multiple countries including Australia [16, 64], Belgium [52], China [69], Egypt [53], Iran [9], Italy [5, 18, 51], Luxembourg [57, 58], Malaysia [31], and the United Kingdom [54], indicating partial acetylation.

The GC‐MS results identified several impurities in heroin samples and are given in Table 2 along with their frequency of appearance and percentage. 6‐MAM is the most observed impurity in street heroin seized from the drug market of Pakistan. It was observed in 97.02% of the total samples analyzed. ACC was observed in 95.46% of the heroin sample analyzed. This high frequency is consistent with international findings where both 6‐MAM and ACC are regarded as key process impurities, often co‐existing with heroin in seized materials.

If we look at the presence of heroin in the suspected powders, 93.26% of the total samples showed positive results, confirming diacetylmorphine. Among the heroin‐positive samples, the concentration of heroin (based on peak area) varied substantially. Three samples were found to be the purest as they did not contain any adulterants. These samples were constituted with COD, ACC, 6‐MAM, heroin, and PAP.

A total of 35 samples tested negative for heroin; however, their chemical composition resembled closely the heroin‐positive samples because they still contained commonly encountered adulterants and impurities. Out of 35, there were 21 samples that showed the presence of 6‐MAM and other poppy alkaloids indicating that acetylation was not carried out completely to produce heroin or degradation from heroin to 6‐MAM might occur during poor storage conditions [73]. However, 14 of the total 35 samples were negative for all alkaloidal impurities indicating that these samples were simply mixtures of adulterants.

### 3.7. Correlation of Common Constituents in Street Heroin and Comparison With Other Studies

**FIGURE 6:**
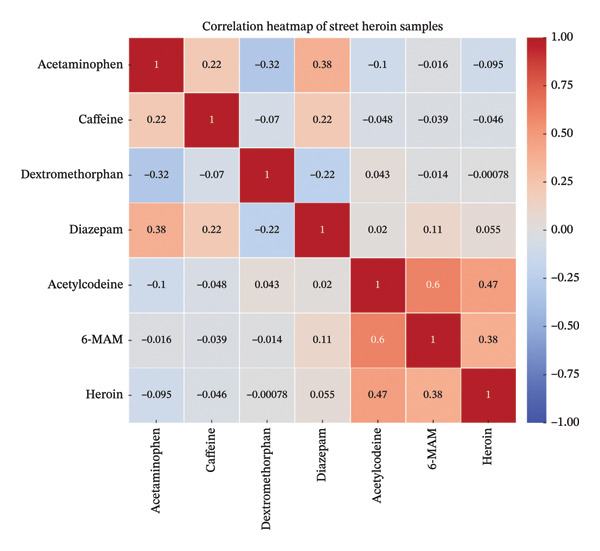
Correlation of the most common adulterants and impurities in street heroin, based on Pearson’s correlation coefficient.

Pearson’s correlation coefficient was used to measure the linear relationship between the most common constituents encountered during analysis, i.e., ACC, ACE, CAF, DEX, DIA, 6‐MAM, and heroin. These correlation coefficient values “r” for each component were used to create a heatmap to visually represent the strength of correlations among them in the illicit street powder (Figure 6). In Figure 6, dark red areas represent strong positive correlations, while dark blue areas show stronger negative correlations. Each cell in the heatmap represents the “r” value for the given pair of components, and it shows how strongly they are linearly related. ACE and DIA exhibit a moderate positive correlation (0.38), and this indicates that these substances are likely to co‐occur as adulterants. Additionally, a strong positive correlation (0.6) exists between ACC and 6‐MAM, suggesting that these impurities are frequently present together, and this is because both are byproducts produced during heroin synthesis. Heroin itself shows a moderate correlation with both ACC (0.47) and 6‐MAM (0.38), reinforcing that these substances co‐exist in many samples.

**FIGURE 7:**
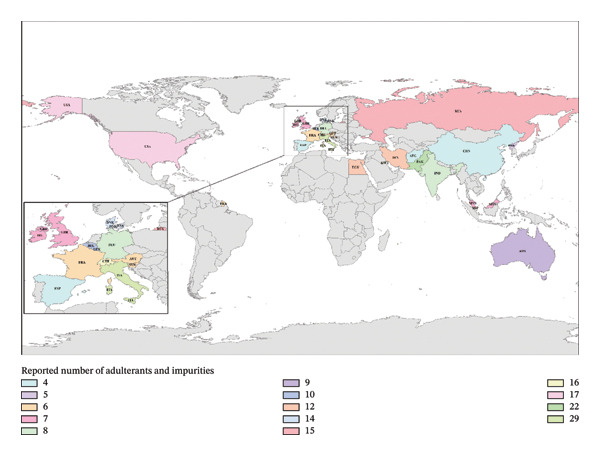
Comparative adulterants and impurities reported in various countries.

A comparison of impurities/adulterants from this study has been carried out with various other countries’ reported data [5, 7, 9–16, 18, 31, 35, 40, 51–67, 69–71] as given in Table 3, which showed a mix of pharmaceutical drugs (analgesics, antimalarials, stimulants, etc.) as adulterants as shown in Figure 7. The highest number of these adulterants/impurities has been reported by Switzerland, the United States, and Italy, i.e., in the range of sixteen to twenty‐nine, and the lowest were reported by Afghanistan and Spain, i.e., four. Previous studies in Pakistan reported four adulterants and four impurities [40, 44], but in this study, fourteen adulterants and eight impurities were found. Across other countries, a varying overlap with Pakistan’s adulterant profile was found. For example, ACE and CAF, both analgesic/stimulant compounds, are present mostly in other countries samples (19 and 21 countries, respectively) as they are in Pakistan. Several opiate‐related compounds (COD, 6‐MAM, MOR, NOS, and PAP) also appear frequently in both Pakistan and many countries. Conversely, certain Pakistan adulterants (e.g., KET, CLO, ETR, HCD, HCT, and LEV) do not appear in any other country’s entries, making them unique to the Pakistan sample. Also, TRA is only found between Pakistan and Ireland, and nicotinamide is found between Pakistan and Germany’s profile. Impurity‐wise, Pakistan’s entry lists no sugars (due to the limitation of GC‐MS technique) or chemical impurities, whereas a few countries do, e.g., Egypt lists ephedrine as an impurity, Switzerland lists gluconic acid, sucrose, and fructose (sugar‐related compounds), the United Kingdom lists strychnine, and the United States lists diltiazem, dipyrone, levamisole, quetiapine, and quinine. These illustrate unique compositional differences (none of which overlap Pakistan).

Chemically, Pakistan’s adulterants cluster in well‐known categories: analgesics/antipyretics (ACE, TRA, etc.), opioids (COD, MOR, NOS, PAP), sedatives/antiepileptics (DIA, PHE, etc.), and stimulants (CAF). Similar categories recur in other countries lists. For instance, analgesics, such as ACE and COD, were also commonly found abroad. Likewise, stimulants especially CAF are ubiquitous. In contrast, compounds without counterparts in Pakistan hint at different adulteration trends. Some countries have antihistamines (chlorpheniramine in Egypt) or psychedelics not seen in Pakistan’s list. In summary, Pakistan’s first entry shows substantial overlap with many countries (e.g., Iran, Italy, and the United States share large subsets of the same adulterants) but also some unique components.

### 3.8. Impacts of Adulterants and Purity of Heroin on the Health of Abusers

The presence of adulterants in heroin is a significant public health concern. CAF is a common adulterant in heroin and is used to increase the drug’s potency and its uptake [7, 74]. However, at high dose, it can also cause adverse effects, such as headache, anxiety, tremors, metabolic acidosis, and heart palpitations [75, 76]. ACE is often added to heroin to give it a powdery texture, to increase its weight, and it is cheap and has a similar melting point to that of heroin [18]. However, it can cause liver failure and intranasal necrosis when taken in high doses [75, 77]. DIA is a benzodiazepine commonly used to treat anxiety and insomnia. It is often added to heroin to enhance its sedative effects. However, it can also cause respiratory depression. DEX is a cough suppressant that is sometimes added to heroin to create a more potent drug. It can cause dizziness, nausea, agitation, and dissociative hallucinations [75, 78]. CHQ is an antimalarial drug, and it can cause a range of adverse effects, including cardiovascular toxicity and retinopathy. CLO is an antifungal drug and is added to heroin. It can cause skin irritation [79, 80]. PHE is a barbiturate that is occasionally added to heroin to enhance its sedative effects. At high dose, it can cause respiratory depression and coma and affect the cardiovascular system [75, 81, 82]. NIC is a form of vitamin B3 that has been found in street heroin. It is not clear why this substance is being added to heroin, but it can cause flushing and itching [83]. The studies on mortality due to drug overdose have shown that the pharmacologically active adulterants have synergistic effects in addition to drug overdose on the central nervous system and cardiovascular system [84–87].

In addition to the pharmacologically active adulterants, illicit heroin is also susceptible to the invasion of various microorganisms. The presence of these microbes can be attributed to the unhygienic conditions that exist in the clandestine laboratories, where these drugs are manufactured. As a result, these drugs and adulterants not only induce their intended psychoactive effects but also have the potential to cause severe health complications due to the presence of harmful microbes. These contaminants may cause infections, which can be particularly hazardous for individuals with compromised immune systems or those who are already suffering from an existing illness [70].

## 4. Conclusions

In conclusion, the street heroin available in Pakistan’s local drug market is adulterated with various pharmaceutical drugs including ACE, CAF, DIA, DEX, CHQ, CLO, NIC, ETR, KET, TRA, CHP, PHE, LID, and LEV. These pharmacologically active substances in street heroin are added during the manufacturing process to increase the bulk and enhance the potency of heroin. The presence of these drugs alone or in combination with others makes it difficult to measure the potency of the drug accurately and increases the threat of drug toxicity or overdose that can be lethal [20]. Their presence highlights the need for increased regulation and monitoring of pharmaceutical drugs in Pakistan to prevent the uncontrolled sale of adulterants. A frequency analysis among various countries underscores the trends that CAF is the most frequent adulterant overall (present in 21 other countries), followed by ACE (19 countries) and various opioids (e.g., codeine or its derivatives in 10–16 countries). In contrast, Pakistan’s exclusive adulterants (KET, CLO, etc.) occur zero times in other countries. This suggests that while Pakistan’s sample shares many common adulterants with global samples (especially analgesics and stimulants), it also contains several rare or unique drugs. The similarity between Pakistani and regional heroin adulteration profiles underscores the transnational nature of illicit heroin production networks. Continuous surveillance and reporting analysis results are essential to identify emerging adulterants.

Having knowledge about the purity of drugs and the presence of adulterants is crucial for policymakers, addiction treatment centers, and physicians. The study emphasizes the importance of implementing validated and standardized procedures to systematically analyze the quantitative analysis of heroin and adulterants present in seized street drugs. The presence of KET, CLO, ETR, HCD, HCT, and LEV as an adulterant in heroin seized from the Pakistani drug market, which appear to be absent in currently available reports from other countries, also deserves further study. The limitation of this study is that the results are based on qualitative analysis with GC‐MS, and no further quantifications were performed. Heroin adulterant cluster analysis could provide important epidemiological and social information concerning those abusing illegal drugs, as performed by other countries.

## Funding

Open access publishing facilitated by Universita degli Studi di Foggia, as part of the Wiley ‐ CRUI‐CARE agreement.

## Conflicts of Interest

The authors declare no conflicts of interest.

## Supporting Information

The Supporting Information associated with this manuscript is provided as Excel files. “Supporting Table S1”, which includes list of certified reference materials and samples used for method validation. “Supporting Table S2”, which includes raw peak areas (signal intensities) for all analyzed samples. Each row represents a different sample, and each column provides the peak area for a specific substance. “Supporting Table S3”, which includes the calculations for Pareto analysis (80/20 rule).

## Supporting information


**Supporting Information** Additional supporting information can be found online in the Supporting Information section.

## Data Availability

The data that support the findings of this study are provided in the manuscript and supporting information.
